# A cross-sectional serological study of bats in the United States Virgin Islands during 2019 to 2020 reveals no evidence of rabies virus exposure

**DOI:** 10.1038/s41598-026-42571-3

**Published:** 2026-03-05

**Authors:** A. Springer Browne, Hannah M. Cranford, Danielle Fibikar, Clint N. Morgan, James A. Ellison, David Horner, Valicia J. Burke-France, Cosme Harrison, Marissa Taylor, Pearl S. Cales, Claudia D. Lombard, Leanne Jankelunas, Nicole F. Angeli, Jenn Valiulis, Jeffrey B. Doty, David J. Worthington, Thomas Kelley, Bethany Bradford, Joseph Roth, Kristine M. Bisgard, Brett R. Ellis, Renata Platenberg, Esther M. Ellis, Ryan Wallace

**Affiliations:** 1 Epidemic Intelligence Service, Division of Workforce Development, National Center for State, Tribal, Local, and Territorial Public Health Infrastructure and Workforce, Centers for Disease Control and Prevention, Atlanta, GA USA; 2 US Virgin Islands Department of Health, Christiansted, VI USA; 3 Domestic Animal Health Analytics Team, United States Department of Agriculture, Fort Collins, CO USA; 4 Homestead Veterinary Services LLC, Oak Creek, CO USA; 5 College of Staten Island, New York, NY USA; 6 Poxvirus and Rabies Branch, Division of High-Consequence Pathogens and Pathology, National Center for Emerging and Zoonotic Infectious Diseases, Centers for Disease Control and Prevention, Atlanta, GA USA; 7 National Park Service, St. John, VI USA; 8 US Fish & Wildlife Service, Sandy Point Wildlife Refuge, St. Croix, VI USA; 9 Division of Workforce Development, National Center for State, Tribal, Local, and Territorial Public Health Infrastructure and Workforce, Centers for Disease Control and Prevention, Atlanta, GA USA; 10 Department of Planning and Natural Resources, Division of Fish & Wildlife, St. Croix, VI USA; 11 Department of Planning and Natural Resources, Division of Fish & Wildlife, St. John, VI USA; 12 St. Croix Environmental Association, St. Croix, VI USA; 13 US Virgin Islands Department of Agriculture, Kingshill, VI USA; 14 University of the Virgin Islands, Charlotte Amalie, VI USA

**Keywords:** Bats, Caribbean, One-Health, Rabies, US Virgin Islands, Zoonoses, Diseases, Ecology, Ecology, Microbiology, Zoology

## Abstract

Bats are a known reservoir of rabies virus in 10 Caribbean nations. A cross-sectional survey of five species of native bats on U.S. Virgin Islands of St. Croix, St. John, and St. Thomas was performed during September 2019–January 2020. Serological testing was used to determine whether native bat populations have been exposed to rabies virus. Bats (n = 72) from seven sampling locations in the United States Virgin Islands (USVI) all tested negative for rabies neutralizing antibodies. The sensitivity of detection of rabies virus antibodies ranged from 49.4% to 100%, depending on geography, bat species clustering, and presumed prevalence. A previous rabies cross-sectional survey conducted during 2019–2020 led by the USVI Department of Health determined freedom-from-rabies for small Indian mongoose (*Urva auropunctata*) populations in U.S. Virgin Islands. These findings, along with a historical absence of rabies detections in passive surveillance of dogs, cats, and wildlife, supports an evidence base that USVI may be rabies-free. These surveillance activities built local One Health capacity and fostered federal, university, and territory collaboration in USVI. Continued surveillance will help determine a declaration of rabies freedom for USVI.

**Supplementary Information:**

The online version contains supplementary material available at 10.1038/s41598-026-42571-3.

## Introduction

**Fig. 1:**
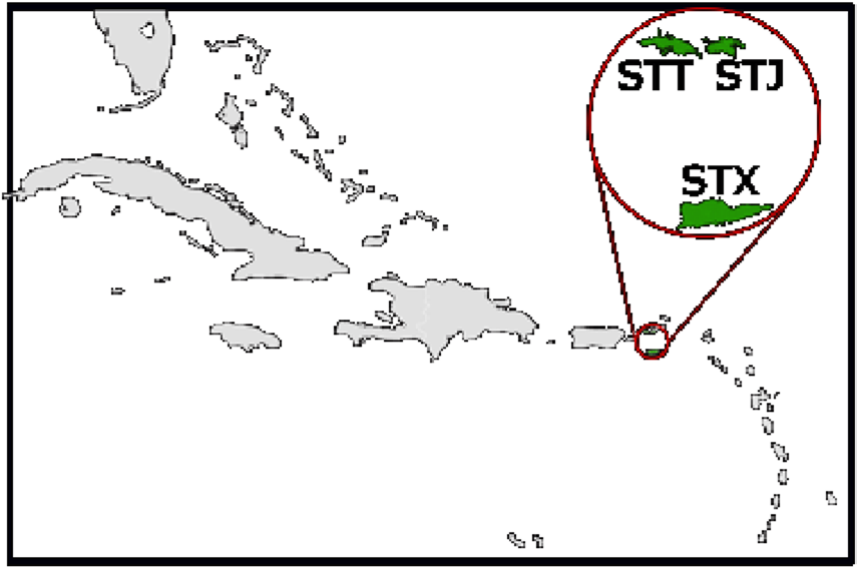
Location of U.S. Virgin Islands (*) in the Caribbean^2^. Asterisk: STX: St. Croix; STJ: St. John; STT: St. Thomas

The rabies virus infects the nervous systems of mammals, resulting in a mortality rate of > 99% in the absence of appropriate post-exposure prophylaxis (PEP). While the virus is prevalent in 10 Caribbean nations where dogs, mongooses, and bats serve as reservoirs^1^, there has not been documented detection of rabies virus in the United States Virgin Islands (USVI). This United States territory encompasses St. Croix, St. John, and St. Thomas Islands and spans 344 km^2^ with an approximate population of 100,000 people, as illustrated in Fig. 1.

In USVI, rabies is categorized as a Class A notifiable disease, mandating immediate reporting to the local Department of Health in case of suspected infection in humans or other mammals. The USVI Department of Agriculture oversees the importation of domestic animals, poultry, and livestock, and rabies vaccinations are required for import of dogs and cats. Suspected rabies cases in any animal, whether domestic or wild, prompt the submission of brain stem and cerebellum samples to the Centers for Disease Control and Prevention for testing. Despite this ongoing passive surveillance, fewer than ten samples are tested annually and rabies has never been identified in any animals (B. Bradford, personal communication).

Rabies has been detected in bats in the Caribbean. Trinidad’s vampire bat (*Desmodus rotundus*) population is a known reservoir for rabies virus, with phylogenetic analysis suggesting three separate introductions from South America^3^. In Grenada, 15.4% of Jamaican fruit bats (*Artibeus jamaicensis*) sampled (n = 52) were exposed to the rabies virus but the virus was not detected in tissues^4^. Rabies exposure was also identified in *Brachyphylla cavernarum* (Antillean Fruit-eating Bats) from a single cave in Puerto Rico during 2012–2014, but similar to Grenada, the rabies virus has yet to be isolated from a bat^5^.

Given the endemic nature of rabies in 10 Caribbean nations, the USVI Department of Health collaborated with local and federal entities to enhance surveillance of rabies within USVI across domestic animal and wildlife populations. Established guidelines from the Pan American Health Organization, CaribVET^6^, and the Office International des Epizooties (WOAH) Terrestrial Animal Health Code, Standards for Animal Health Surveillance (Chapter 1.4)^7^ were used as reference for the study design. A scientifically based prospective cross-sectional survey during 2019–2020 obtained statistically significant sample size to determine the absence of rabies in small Indian Mongoose (*Urva auropunctata*) in USVI^2^.

Five bat species are native to the USVI (Table 1) and with diets of primarily insects, fish, fruit, and nectar. Puerto Rico is approximately 60 km from St. Thomas in USVI, therefore a risk of rabies virus spillover from the Puerto Rican bat population into the USVI bat population exists. Furthermore, human-mediated movement of animals (including bats) and natural weather events such as hurricanes pose a continuous threat for the introduction of rabies into USVI bat populations. We describe a prospective cross-sectional study to determine the absence of rabies in bat populations in the USVI.

**Table 1:** Characteristics of bat species in U.S. Virgin Islands.

Species	Common name	Diet	Habitat
*Molossus molossus*	Pallas’ Mastiff Bat	Insects	Open
*Noctilio leporinus*	Greater Bulldog Bat	Fish/Insects	Water
*Brachyphylla cavernarum*	Antillean Fruit-eating Bat	Fruit/Nectar/Insects	Narrow/Forest
*Artibeus jamaicensis*	Jamaican Fruit-eating Bat	Fruit/Nectar/Insects	Narrow/Forest
Stenoderma rufum*	Red Fig-eating Bat	Fruit/Nectar/Insects	Narrow/Forest

*Stenoderma rufum has rarely been observed in the U.S. Virgin Islands^8^.

## Materials and methods

This survey was conducted in parallel with a similar freedom-from-rabies study of mongooses in USVI^2^; the ethics, permits, and study design are identical between the two studies and briefly described below.

### Ethics and permits

All sampling was carried out with relevant guidelines and regulations for animal sampling, including ARRIVE guidelines (https://arriveguildlines.org). The CDC Institutional Animal Care and Use Committee (IACUC) approved all animal sampling procedures under protocol number 2929DOTMULX-A5. Additionally, the CDC confirmed that this project was exempt from Human Subject Research protocol review. Necessary sampling permits were secured from the National Park Service (Permit #VIIS-2019-SCI-0028), the US Fish & Wildlife Service (Sandy Point National Wildlife Refuge Research and Monitoring Special Use Permit #2019-005), and the USVI Department of Planning and Natural Resources (Permit #DFW19049U). All individuals handling bats were vaccinated with one of two FDA-approved rabies vaccines (IMOVAX, RabAvert) within a year prior to fieldwork. Titer checks were conducted on all local collaborators who had received vaccination over a year before the commencement of fieldwork.

### Study design

Our research objective was to determine freedom-from-rabies for bats in USVI. In order to detect rabies in the bat population in USVI, we assumed a conservative rabies sero-prevalence of 10%, based on other Caribbean studies^3–5^. The primary sampling unit for this surveillance project was the population of a particular species, within a specific region, based on contiguous plots of forest within each island, and wetland/coastal habitats (Supplemental Fig. 1). Sampling in each of these small regions (approximately 8500 acres each) allows for increased sensitivity of detection with smaller population clusters tested.

To determine the required sample size, EpiTools FreeCalc was used^9^. This method factors in population size, presumed prevalence, and the sensitivity and specificity of the screening test, compensating for increased sampling needs because of imperfect test accuracy^10^. These calculations are based on Bayesian techniques that consider the sensitivity (100%) and specificity (98.34%)^11^ of the Rapid Fluorescent Foci Inhibition Test (RFFIT) applied to serum samples^10^. The RFFIT specifically identifies rabies virus neutralizing antibodies in serum, indicating prior exposure to the rabies virus antigen. All laboratory analyses were conducted at CDC’s National Rabies Reference Laboratory, an accredited reference laboratory by the World Organization for Animal Health.

Based on sample size analyses, a minimum target sampling of 24 bats per species in each of the ten regions was established, with at least two sampling sites selected per region to increase detection sensitivity, for an objective of 960 bats sampled. Bat sampling sites were preselected based on geographic distribution, preferred habitats (Table 1), and known high density bat roosts identified by local collaborators.

### Sampling

Bats were live-caught using mist nets setup in the early evening, and were placed in a sampling bag. One field sampling location (Abandoned Rum distillery, St. Croix Island) allowed use of a hand net for capture of bats, due to the high density of bats in the roost. Bats were manually restrained for sampling, and then released back in the wild.

A 30 gauge needle was used to pierce the cephalic vein, and a Sarstedt MicrovetteÒ 100 Z or 200 Z was used to collect blood. The blood was later centrifuged at 2000 G for 15 min for serum analysis. No more than 1% of a bats’ body weight of blood was collected, in accordance with veterinary standards for exotic species and wildlife. Given the average weight of the smallest bat species native to USVI (*Mollosus mollusus*) is approximately 10g, the maximum collection was 100 uL to 200 uL, depending on the weight of the species.

### Serology

RVNA titers were determined by the RABV micro-neutralization test, which was developed to test smaller volumes of serum and is used as a comparable assay to the traditional rapid fluorescent focus inhibition test (RFFIT). The micro-neutralization test was conducted following the protocol described by Smith and Gilbert^12^. The RVNA titers of individual bats were calculated by the Reed-Muench method and were converted to international units (IU/mL) by comparison to a standard rabies immune globulin (SRIG) control containing 2 IU/mL^13^. For the objective of this study, positive RVNA titers (≥ 0.1 IU/mL) were defined by at least 50% neutralization of the RABV challenge virus dose (50 focus-forming doses). All serum samples were initially tested at a 1:10 dilution. Final titers less than 0.1 IU/mL were considered negative for the presence of RVNA for the purposes of this investigation.

### Analysis

Statistical analyses were performed using EpiTools 1-stage freedom analysis^9^ based on a method a binominal distribution with known specificity and sensitivity of our serology tests^14^. Various design prevalence values (i.e. multiple presumed rabies prevalence proportions of the sampled population) were assessed, with clustering of populations varied between species, island, and entire population. A cut-off point of zero was used (i.e. if one positive was detected, the population would not be disease-free). Model outputs determine sensitivity of detection of disease (i.e. confidence of the determination of disease freedom based on the inputs).

A literature review of rabies and lyssavirus serology research since 2010 was performed to evaluate known seroprevalence of rabies and lyssavirus in bats throughout the world (Supplemental Table 1). These values were used to calculate quartile and median estimates of rabies virus prevalence values in the Americas and the Caribbean to calculate the sensitivity of rabies virus freedom.

## Results

Out of fifteen sampling events, four yielded no successful bat captures. An average of 5.7 bats were captured per sampling event, with the largest number (n = 21) captured in a single day with hand nets at the Abandoned Rum Distillery on St. Croix Island (Table 2). Supplemental Table 2 reveals the sex and average weight of adult bats sampled during the study.

**Table 2:** Counts of bats tested for rabies neutralizing antibody by species and island in U.S. Virgin Islands, September 2019–January 2020 (n = 72).

Island	Total sampled	Sample site	Antillean fruit-eating bat	Greater bulldog bat	Jamaican fruit-eating bat	Pallas’ Mastiff bat
St. Croix	30	Abandoned Rum Distillery	27		3	
St. John	19	Fish Bay Gut				12
		Lameshur Bay	1	2		1
		Reef Bay			3	
St. Thomas	23	Dorothea		13		
		Magen’s Bay		1	4	3
		Neltjeberg Bay		2		
Total	72	US Virgin Islands	28	18	10	16

No bats tested positive for rabies virus neutralizing antibodies.

**Fig. 2:**
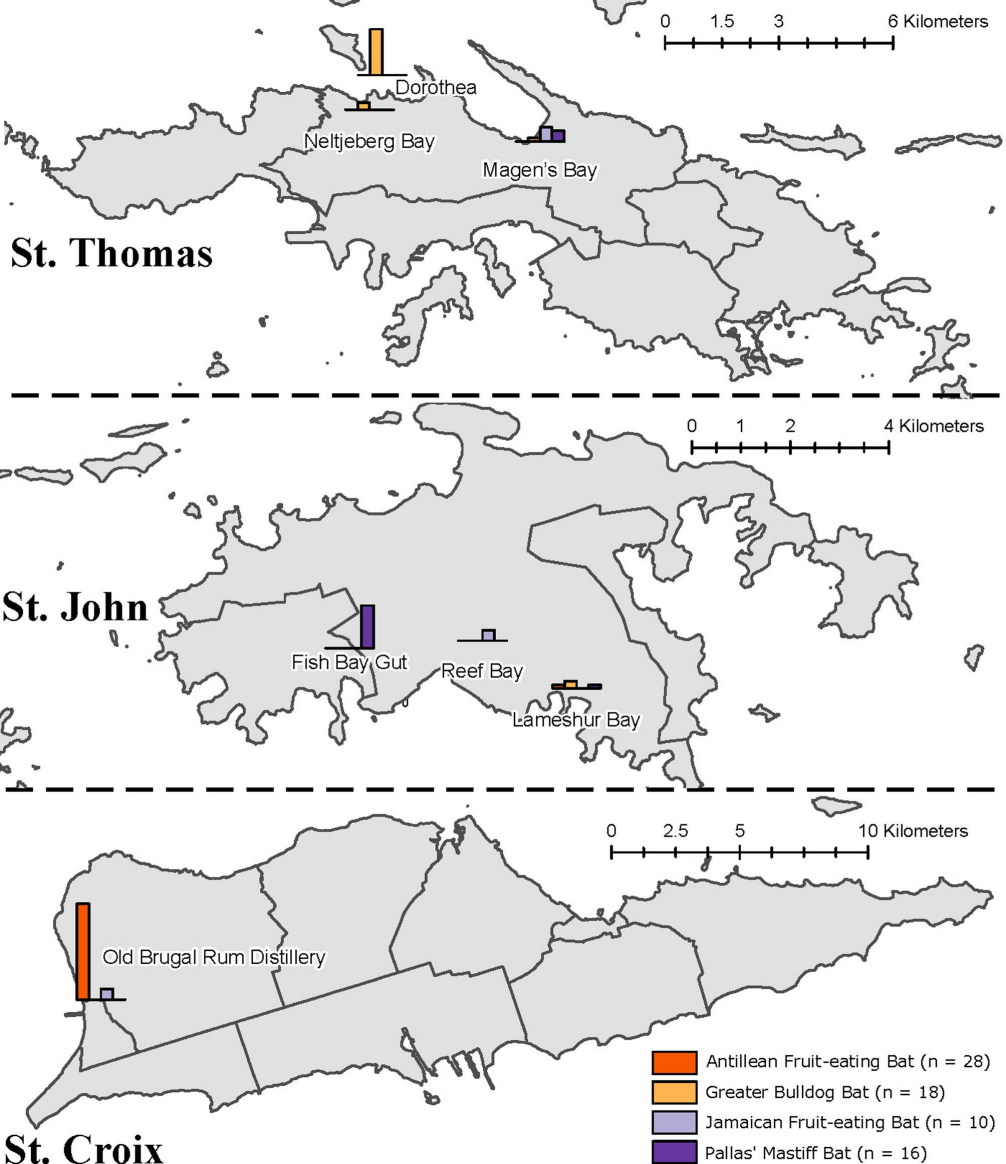
Number of bats sampled and tested for exposure to rabies virus (serology) — United States Virgin Islands, September 2019–January 2020 (*, ±). Asterisk: no bats tested positive for rabies virus neutralizing antibodies (n = 72). Plus or minus: map generated using ArcGIS Pro 3.1 https://www.esri.com/en-us/arcgis/products/arcgis-pro/overview.

Bats were sampled (n = 86) at 7 sampling sites across USVI during September 2019–January 2020 (Fig. 2); a total of 72 bats had sufficient high-quality serum collected for rabies antibody testing (Table 2), revealing all to be negative for exposure to the rabies virus. Using modelling methods previously described, the sensitivity of detection of rabies, based on our sample size of bats and clustered by island and species, ranged from 49.4% to 100% (Table 3**).**

**Table 3:** Freedom-from-rabies determination sensitivity based on bats sampled (n = 72) in U.S. Virgin Islands, September 2019–January 2020, clustered by island and species.

Cluster	Total sampled (n)	Sensitivity (%) based on seroprevalence		
		5% prevalence	10% prevalence	40% prevalence
Antillean fruit-eating bat	28	85.1	96.7	100
Pallas’ mastiff bat	16	66.3	85.8	99.9
Jamaican fruit-eating bat	10	49.4	70.5	98.3
Greater bulldog bat	18	70.6	88.9	99.9
St. Croix	30	87.0	97.4	100
St. John	19	72.5	90.2	100
St. Thomas	23	79.1	99.9	100
All species and islands	72	99.3	100	100

Laboratory test sensitivity (100%) and specificity (98.34%), presumed prevalence (5%, 10%, 40%), population size (infinite), and cutoff detection (n = 1) were used in calculations^9, 14^. Presumed prevalence was roughly estimated based on median and quartile estimates of peer reviewed scientific research of rabies virus serology prevalence in bat species in the Americas and Caribbean (^15–30^), Supplemental Table 2; Q1 7.9%, median 13.65%, Q3 37.9%).

A moratorium on bat sampling because of the COVID-19 epidemic in February 2020 led to a complete cessation of sampling activities before our study design sample size goals were achieved. All USVI Department of Health resources were redirected to mitigate the COVID-19 epidemic.

## Discussion

Our prospective cross-sectional surveillance study revealed no evidence for rabies virus exposure in any bats sampled (n = 72) from USVI, with a sensitivity of detection ranging from 49.4% (Jamaican Fruit-eating Bat) to 97.4% (St. Croix Island) depending on how sample populations were clustered. Our sample number (n = 72) failed to achieve our statistical goal of sampling for the ten regions and four species in USVI; this shortcoming is discussed in explicit detail toward the conclusion.

The absence of rabies virus exposure in bats was suspected, given the lack of detection of rabies infection in clinically suspected USVI domestic animals, and results from a robust prospective cross-sectional study that demonstrated freedom-from-rabies virus by antigen testing and rabies virus exposure by antibody testing in small Indian mongooses^2^. In contrast, in Grenada 15% of sampled Jamaican Fruit-eating bats were seropositive for rabies^4^.

Surveillance of a single bat colony of Antillean fruit-bats in Puerto Rico revealed evidence that these bats are a cryptic reservoir for rabies virus, although no virus has yet been isolated^5^. Transmission of rabies from Puerto Rican bat populations poses a potential risk of rabies incursion for USVI, given the close proximity and frequency of weather events that are known to move animals between islands. Puerto Rico and USVI both host the Antillean Fruit-eating bat, and this species dominates a large colony on St. Croix (i.e. Abandoned Rum Distillery, Table 2). Puerto Rico is also host to a well-known terrestrial rabies reservoir, the small Indian mongooses (n = 16) and frequently reports spillover events into dogs and cats^31^. In Puerto Rico, molecular epidemiology revealed two rabies virus variants circulating in mongooses that are genetically linked to the north central skunk strain which originated in the continental United States, while the source of Cuba’s rabies variants was linked to a Mexican dog rabies virus^32^. Despite their geographic isolation, Caribbean islands are still vulnerable to introduction of rabies virus from mainland areas. These data suggest that despite the geographical isolation of the US Virgin Islands, ongoing import health certification requirements for animals entering USVI are essential to prevent the incursion of rabies virus^33^.

Vampire bats (*Desmodus rotundus*) are a key rabies reservoir host and contribute to human and bovine outbreaks throughout Latin America^34, 35^. In close proximity to the Caribbean, vampire bats are the primary rabies reservoir in French Guiana, Guyana, Trinidad, and Belize; all lie within the continental Americas except Trinidad^1^. In a comprehensive modelling analysis, USVI was ranked as the 3rd least likely Caribbean nation to have rabies virus present in bats, partially because of its lack of Vampire bats and geographic location^34^. While the Caribbean wasn’t included in formal modelling analyses of Vampire bat habitat throughout the Americas, the authors did conclude that suitable habitat exists and risk will increase with more cattle present^36^. If Vampire bats were to become resident in USVI, the risk of rabies transmission would dramatically increase.

While our surveillance methods determined no exposure of rabies virus in the bats sampled, and we were able to determine a sensitivity of 49.4%–100% detection based on species and island and assumed prevalence (Table 3). Some low sensitivities of detection (e.g. 49.4% rabies detection sensitivity in Jamaican fruit-eating bats) would not provide confidence in rabies freedom using this clustering method with only 10 bats sampled; higher sensitivities may provide some confidence of disease absence, but depend on clustering and presumed prevalence. We also did not obtain a sufficient sample size for a blanket determination of freedom-from-rabies for bat populations in USVI. Our rigorous cross-sectional design method using ten regions across three islands was successfully implemented for a determination of freedom-from-rabies for mongoose populations in USVI^2^, but might have been unrealistic for bat surveillance, which requires more sampling effort: four bat species instead of one mongoose species, bats are more challenging to capture, safely sampling bats requires more time and training. Achieving a minimum of 24 samples per species (n = 4 native) for each region (n = 10) would require 960 viable samples. This study experienced a nightly capture rate of 5.7 bats; achieving the a priori study goals would require almost 170 nights of sampling given our capture rate. While our study design was thorough it may present an unrealistic expectation for bat rabies surveillance.

Clustering by island and across species offered more encouraging results (Table 3), but requires assumptions of bats moving between islands and cross-species interactions between bats. Identical rabies virus clades were shared between up to four different bat species in Colorado, indicating active inter-species transmission of rabies occurs between bats^37^. Given Colorado is over 269,000 km^2^, and the total land area of the US Virgin Islands is 344 km^2^, it is reasonable to assume that clustering bat surveillance by island may be a valid approach to disease surveillance due to small geographic area. Migratory behavior is less common in tropical bats^38^, and a sustained food source encourages bats resident on USVI to not migrate. Jamaican fruit bats are known to forage under 500 m from their roosting sites^39^, while the Greater Bulldog Bat stays resident in tree hollows in large colonies^40^, which was observed at the Dorothea sampling site on St. Thomas (Table 2). Six species of bats in Trinidad showed rabies virus seropositivity, including the Jamaican Fruit-eating Bat, native to USVI^41^. While these observations suggest that multiple bats species resident in USVI may form homogeneous populations from a disease perspective, DNA testing of bats would provide more definitive evidence of lack of migration both between islands in USVI, and to other nations in the Caribbean.

A more realistic surveillance approach might be using longitudinal sampling with modelling^10^ to determine freedom from disease. This design has been successfully implemented by the Food and Agriculture Organization to determine the absence of Foot-and-Mouth Disease in the Thrace region (i.e. Greece, Turkey, Bulgaria)^42^. Repeated testing at sentinel sites (e.g. large population roosts on each island such as the abandoned rum distillery on St. Croix) would succeed in monitoring potential rabies incursions into the USVI bat populations, however the cost and human resources to operate such a sampling scheme may not be available. The geographic isolation and relative size of each island (50–220 km^2^ in area) lends itself to a reasonable assumption of mixing of bat populations and detection of exposure if rabies were present if sampling is conducted in a cross-sectional manner (i.e. over a short time period).

In addition to active surveillance utilizing advanced statistical methods, passive surveillance of sick and found-dead bats continues to be a viable method for rabies reporting within USVI. Although only one bat was submitted for necropsy by members of the public during 2018–2020, this is similar to a 0.3 wildlife tested per 100,000 human population per year worldwide^43^. When considering rabies surveillance of all animals, road-side sampling, public reporting due to animal bites, and other found dead animals can be used surpass the global annual median all-animal testing rate of 1.53 animals per 100,000 humans^43^. This noted, a single sample over two years is not sufficient to make a determination of rabies freedom.

Passive surveillance of suspected rabid animals is a cost-effective and targeted approach to increase sensitivity of detection^43^. However, when passive surveillance is not identifying enough samples to meet sample size objectives, more active approaches like this study are required. A One Health approach, with statistically driven active and passive surveillance activities, stringent import restrictions, and public education will provide a framework to decrease the risk of incursion of rabies virus into USVI. While this study did not meet its sample size objectives, there are valuable lessons from this effort. This is the first study to describe a strategic sampling framework to address species clustering, species-specific rabies virus freedom, and geographic disease freedom using sensitivity and specificity of the detection method (i.e. serology). The failure of this study to reach sample size is also a valuable scientific lesson on the complexity and resource-requirements for sampling a species in difficult terrain. Lastly, the lack of finding any antibody in over 70 bats sampled, combined with a lack of historical rabies virus detection, contributes to bat rabies surveillance in the Caribbean. Wildlife, domestic animals, and people will always pose a threat to introduction of rabies virus, necessitating continued animal importation regulations, passive surveillance, and periodic cross-sectional studies as described here. We believe that a sampling framework that prioritizes longitudinal monitoring at sentinel high-density sites, such as the abandoned rum distillery in St. Croix, complemented by strong passive surveillance to detect any incursions promptly, would be a feasible and resource-efficient strategy to monitor for rabies in bat populations.

## Supplementary Information

Below is the link to the electronic supplementary material.

Supplementary Material 1

## Data Availability

Data is provided within the manuscript or supplementary information files.
