# Supplementary material for: A cross-sectional serological study of bats in the United States Virgin Islands during 2019 to 2020 reveals no evidence of rabies virus exposure
Source: Sci Rep. 2026 Mar 5;16:12111. doi: 10.1038/s41598-026-42571-3 (PMC13077009; doi:10.1038/s41598-026-42571-3)
Supplement: Supplementary file 1 — Supplementary Material 1 [file 41598_2026_42571_MOESM1_ESM.docx]

**Supplemental Table 1:** Summary of bat rabies and lyssavirus serological surveillance since 2010 by continent, sample size, and seroprevalence

| **Continent** | **Country** | **Sample size** | **Species** | **Seroprevalence rabies** | **Other lyssavirus** | **Citation** | **Years Sampled** |
| --- | --- | --- | --- | --- | --- | --- | --- |
| **Caribbean** | Trinidad | 383 | 21 species | 8.6% |  | (Seetahal et al. 2020) | 2012-2017 |
|  | Grenada | 111 | *Artibeus jamaicensis*, *Artibeus lituratus*, *Glossophaga longirostris*, *Molossus molossus* | 7.2% |  | (Zieger et al. 2017) | 2015 |
|  | Puerto Rico | 218 | *Brachyphylla cavernarum* | 6.5% |  | (Hirsbrunner et al. 2020) | 2012-2014 |
|  | French Guiana* | 992 | 30 species | 10.1% |  | (de Thoisy et al. 2016) | 2005-2013 |
| **Americas** | Brazil | 1049 | 59 species | 17.5% |  | (Almeida et al. 2019) | 2010-2015 |
|  | Brazil | 230 | 23 species | 64% |  | (Costa et al. 2017) | 2013-2015 |
|  | Brazil | 64 | *Desmodus rotundus* | 25% |  | (Megid et al. 2021) | 2015-2016 |
|  | United States of America | 3198 | *Eptesicus fuscus* | 9.4%-17.9% |  | (O’Shea et al. 2014) | 2001-2005 |
|  | Brazil | 307 | 28 species | 50.8% |  | (Costa et al. 2017) | 2009 |
| **Africa** | Ghana | 304 | *Eidolon helvum* |  | 44.7% | (Suu-Ire et al. 2017) | 2012-2014 |
|  | Anjouan, Mayotte,  La Réunion, Mauritius,  Mahé,  Madagascar | 572 | 22 species |  | 17.9% | (Mélade et al. 2016) | 2010-2015 |
|  | Kenya | 769 | 17 species |  | 6.9% | (Kuzmin et al. 2011) | 2009-2010 |
| **Eurasia** | Bulgaria,  Czech Republic, Poland, Russia Slovenia | 1027 | 25 species |  | 3.2% | (Seidlova et al. 2020) | 2014-2018 |
|  | Poland | 115 | 13 species |  | 30.4% | (Orłowska et al. 2020) | 2012-2018 |
|  | Croatia | 350 | 7 species |  | 5.71% | (Šimić et al. 2018) | 2016-2017 |
|  | France | 214 | Eptesicus serotinus |  | 70% | (Robardet et al. 2017) | 2009-2015 |
|  | Spain | 406 | 9 species |  | 17.5% | (López-Roig et al. 2014) | 2004-2012 |
|  | Finland | 423 | 6 species |  | 1.12%-3.36% | (Nokireki et al. 2013) | 1985-2012 |
|  | Spain | 2,393 | 20 species |  | 20.7% | (Serra-Cobo et al. 2013) | 2001-2011 |
|  | China | 685 | 8 species |  | 2.2% | (Jiang et al. 2009) |  |

***While present on mainland South America, this country is included in previous analyses of prevalence of bat rabies in the Caribbean** (Morgan et al. 2020)

**Supplemental Table 2**: Mean weight in grams of adult bats sampled (n = 60) by species and sex in United States Virgin Islands, September 2019–January 2020*

| **Species** | **Total Sampled**  **(n)** | **Female** | | **Male** | | |
| --- | --- | --- | --- | --- | --- | --- |
|  |  | **Count**  **(n)** | **Mean Weight**  **(g)** | | **Count**  **(n)** | **Mean Weight**  **(g)** |
| Antillean Fruit-eating Bat | 28 | 2 | 49 | | 26 | 50 |
| Greater Bulldog Bat | 7 | 3 | 56 | | 4 | 72 |
| Jamaican Fruit-eating Bat | 9 | 4 | 41 | | 5 | 38 |
| Pallas’ Mastiff Bat | 16 | 11 | 13 | | 5 | 16 |

*Twelve juvenile bats (n = 11 Bulldog bats; n = 1 Jamaican Fruit bat) were excluded from analyses.
